# Structural and *In Silico* Characterization of Small Molecules Isolated from *Eichhornia crassipes*

**DOI:** 10.1155/2020/1375639

**Published:** 2020-05-15

**Authors:** Andrew Mtewa, Duncan C. Sesaazi, Fanuel Lampiao

**Affiliations:** ^1^Chemistry Section, Department of Applied Sciences, Institute of Technology, Malawi University of Science and Technology, Blantyre, Malawi; ^2^Pharmbiotechnology and Traditional Medicine Center (PHARMBIOTRAC), Mbarara University of Science and Technology, Mbarara, Uganda; ^3^Africa Center of Excellence in Public Health and Herbal Medicine (ACEPHEM), College of Medicine, University of Malawi, Zomba, Malawi

## Abstract

*Eichhornia crassipes* has been reported to have various medicinal properties including anticancer activities. The plant was collected from the Shire river in Malawi, and two cytotoxic compounds, benzene-1,4-diol and nonanedioic acid, were isolated and characterized for the first time in the leaves and roots of the plant. ^1^H NMR, COSY, HSQC, HMBC, 13-C, and LCMS spectroscopic experimental techniques were used to identify the compounds in their pure forms. *In silico* experiments showed that both compounds do not have AMES toxicity and do not inhibit cytochrome P450 enzymes, but nonanedioic acid acts as a CYP2D6 substrate. This work showed that *Eichhornia crassipes* can be considered to have a role as a source of potential hits and leads to drug development that can be rationally optimized for drugs.

## 1. Introduction

Small molecules are basically molecules with low molar masses. Over the years, they have been found to be of much use in drug designing and development due to their small sizes that enable them to manoeuvre through membranes with relative ease (save other properties). They have fairly high selectivity of substrate and high binding energy [1, 2]. Small molecules have also been reported to make up the whole lot of traditional drugs and a majority of other therapeutic drugs (over 90%) that have been and are currently in the market [3]. The magnitude of the size of a drug entails the extent to which it will be interacting with membranes, receptors, and other proteins and substrates throughout its kinetic and dynamic lifetime, guaranteeing its delivery to their biological targets. Small molecules have been reported to be significantly useful in designing drugs against various diseases such as cancers [4–6]. They appear to remain a formidable platform for drug designing due to their high efficiency in the fight against parasites such as viruses, bacteria, and fungi. The development of drugs from small molecules can be done by metabolizing the molecules, but largely, it is done through a rational build-up process where small bits of molecules are added to the active small molecule creating a series of compounds that can be screened and assessed for their bioactivities and pharmacokinetic and pharmacodynamic properties [7–9]. Although most times drug potency is placed above its chemistry and pharmacokinetics [10] particularly in most natural products in traditional medicine, a successful drug cannot be developed by one without the other. Research on plant-based drug development focussed on clinical aspects of the development process is still lacking particularly in the developing world [5]. There is therefore a current need for a systematic rational balance of various properties in developing drugs from plants. Plants have contributed a lot to various conventional drugs over the years. One of such plants is *Eichhornia crassipes* (namasipuni). This is a common weed that grows in water and often proliferates on the surfaces of water bodies. In some parts of Malawi and Uganda, the plant is used to treat chronic wounds and cancers through both local and oral administration. The plant has been reported to have medicinal properties including antioxidant, anticancer [10, 11], diuretic, antiarthritic, antimicrobial, anti-inflammatory, nematicidal, hepatoprotective, antiasthmatic, hypocholesterolemic, antiacne, antieczemic, anticoronary, hemolytic, antiandrogenic, and antihistaminic activities [12–16] which provide a significantly important source of potential drug hits and possibly leads against various diseases and malmanifestations including cancers. According to a systematic review conducted in 2018 on the plant, more research on the isolation and characterization of compounds of the plant is warranted. However, it is also noted that compounds elucidated this far were isolated from the leaves and the roots which needed to be explored as well.

## 2. Materials and Methods

### 2.1. Reagents and Materials

Ethyl acetate, methanol, dichloromethane, and n-hexane were purchased from Sigma-Aldrich (Germany). Thin-layer chromatography (TLC-F_254_) was obtained from Merck (Darmstadt, Germany) and LCMS from Agilent technologies (California, USA). StarDrop® (version 3.4) was obtained from Optibrium Ltd., and the pkCSM ADMET descriptors algorithm protocol was accessed from the University of Cambridge online [17].

### 2.2. Plant Collection and Identification

Whole plants of *Eichhornia crassipes* were collected from Liwonde, Malawi, in the Shire river at the following GPS coordinates: 15°03'12.9”S 35°13'13.2”E, 15°03'24.9”S 35°13'13.4”E, 15°03'11.3”S 35°31'12.5”E, and 15°03'07.5”S 35°13'15.4”E between February and March of 2019. The identification of the plant was done by Mr. Hassam Patel of the Malawi Herbarium and Botanic Garden (MHBG). The plant can be accessed from the MHBG under accession number 35964.

### 2.3. Preparation of Plant Material

The plant was prepared as described in the literature [11, 18] with minor changes suitable for the conditions and solvents available. The plant material was washed continuously using tap water and then distilled water. The samples were air-dried at room temperature in a ventilated room for about 35 days, ground to powder, and then kept at −10°C for subsequent use. Extraction of the material (about 500 g) was done three times in methanol (2.5 L) over a 24-hour period in a reduced light environment cupboard. The methanol extracts of the plant were dried at 40°C *in vacuo*.

### 2.4. Isolation, Purification, and Mass Determination

About 2 g of the dried extract was run in a manual column as follows: hexane : ethyl acetate (10 : 0, 9.5 : 0.5, 9.0 : 1.0, 8.5: : 1.5, 8.0 : 2.0, 7.5 : 2.5, 7.0 : 3.0, 6.0 : 4.0, and 5.0 : 5.0) and dichloromethane : methanol (10.0 : 0, 9.5 : 0.5, 9.0 : 1.0, and 8.5 : 1.5). Purification was being monitored using silica gel precoated thin-layer chromatography (TLC-F_254_) under UV (254/365 nm) and normal light. Fractions with single spots and similar mobile rates were combined, and where possible, impure fractions with more than one spot were separated further to a much smaller scale with a 10 ml volume syringe column using appropriate solvent systems as guided by TLC profiles. Percentage yield was determined from the mass of the dry starting material used. A liquid chromatography mass chromatography (LCMS, Agilent) UV detector was used to confirm purity (single peaks) as well as the molar mass of the pure compounds isolated. The LCMS was run with 5–95% acetonitrile/water and +1% formic acid as described in the literature [2] from 100 to 1300 m/z range.

### 2.5. Determination of Drug Pharmacokinetic and Pharmacodynamic (PKPD) Properties of the Isolated Compounds

PKPD properties of the compounds were determined *in silico* from the structures obtained through a combination of 2D spectroscopic and chromatographic techniques. Structures were designed and run in StarDrop® (version 3.4; Optibrium Ltd.), and additional information was determined by running the simplified molecular-input line-entry system (SMILES) of the structures on the pkCSM ADMET descriptors algorithm protocol platform as explained in the literature [19].

## 3. Results

**Figure 1:**
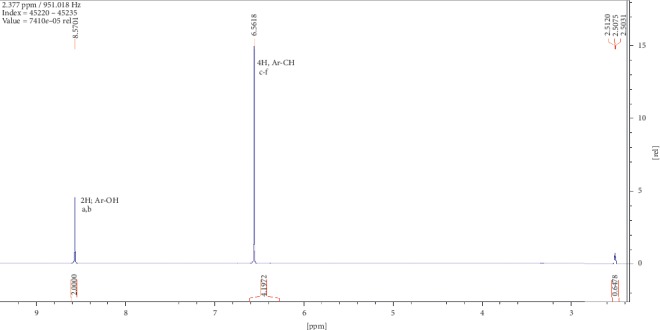
Proton nuclear magnetic resonance spectroscopy profile for compound (1).

**Figure 2:**
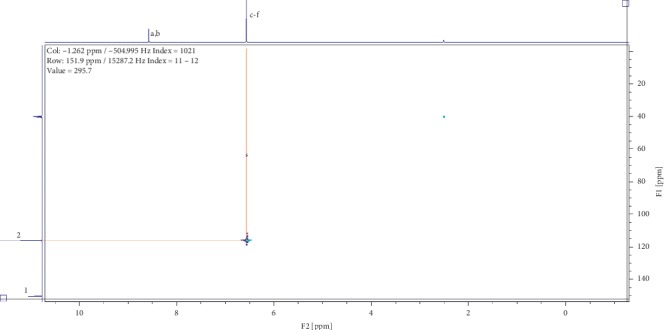
Heteronuclear single quantum coherence spectroscopy profile for compound (1).

**Figure 3:**
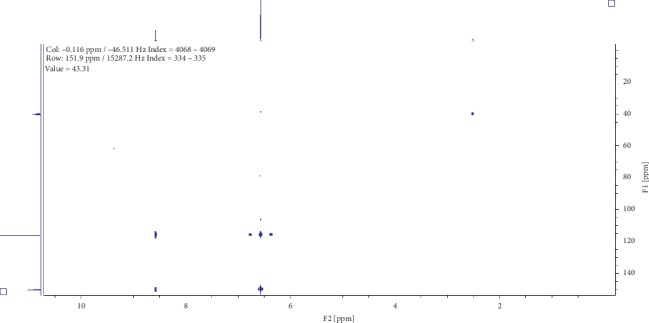
Heteronuclear multiple bond coherence profile for compound (1).

**Figure 4:**
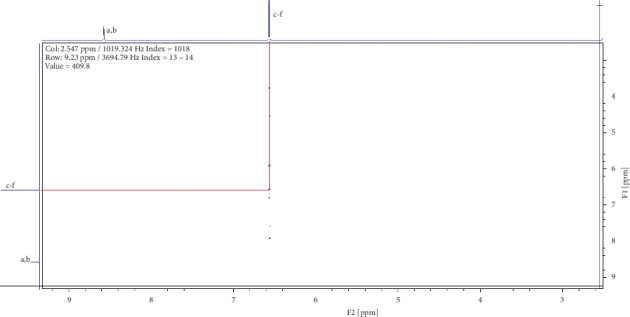
Proton-proton correlated spectroscopy profile for compound (1).

**Figure 5:**
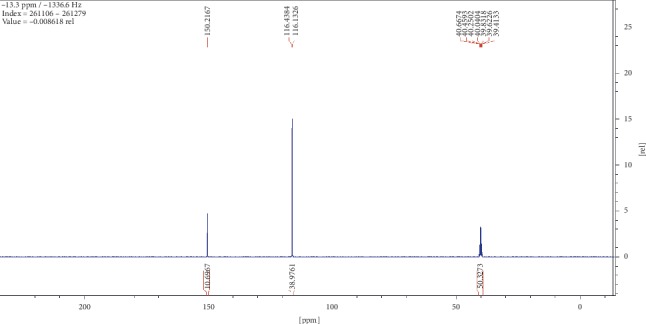
13-carbon spectroscopy profile for compound (1).

Two compounds were elucidated, one from the roots and the other from the leaves of *E. crassipes*. Compound (1) was obtained as a white powder with ^1^H NMR profile (500 MHz, DMSO; *δ* ppm) as follows: *δ*8.57(2H*, s, Ar-OH*) and *δ*6.57(4H, *s, Ar-CH*) and 13-C (500 MHz, DMSO; *δ* ppm) as follows: *δ*150.2 and *δ*116.1. Figures 1–5 show proton nuclear magnetic resonance spectroscopy (^1^H NMR), heteronuclear single quantum coherence spectroscopy (HSQC), heteronuclear multiple bond coherence (HMBC), proton-proton correlated spectroscopy (COSY), and 13-carbon spectroscopy profiles of compound (1).

**Figure 6:**
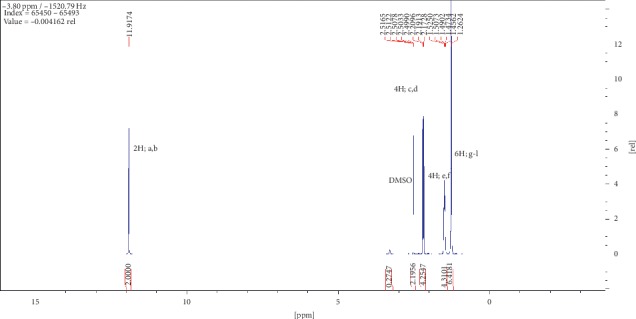
Proton nuclear magnetic resonance spectroscopy profile for compound (2).

**Figure 7:**
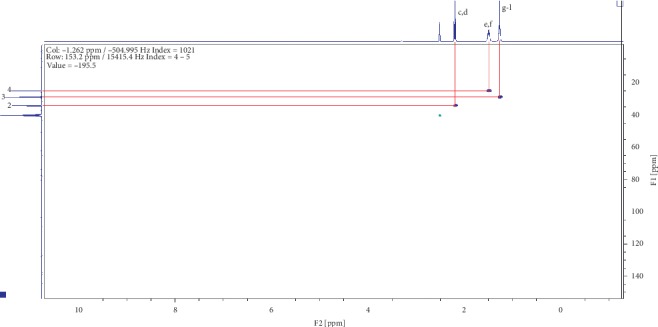
Heteronuclear single quantum coherence spectroscopy profile for compound (2).

**Figure 8:**
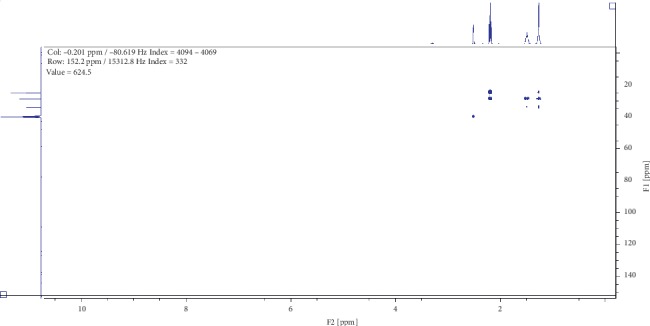
Heteronuclear multiple bond coherence profile for compound (2).

**Figure 9:**
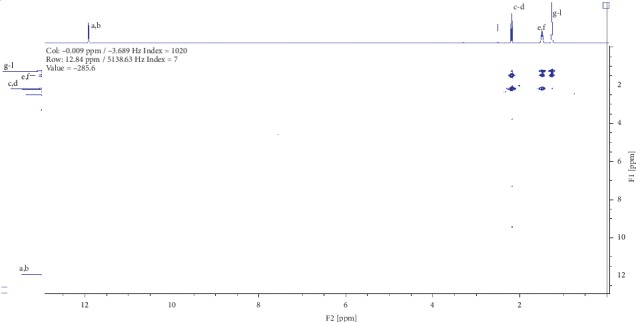
Proton-proton correlated spectroscopy profile for compound (2).

**Figure 10:**
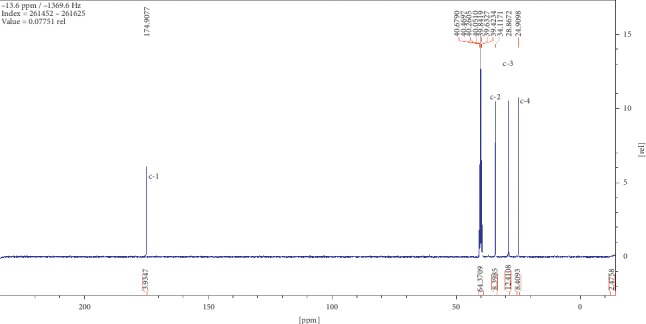
13-carbon spectroscopy profile for compound (2).

Compound (2) was obtained as a white powder with ^1^H NMR profile (500 MHz, DMSO; *δ* ppm) as follows: *δ*11.92 (2H,*s,C(CO)OH*), *δ*2.19 (4H, *t, J*7.3*, CH2*), *δ*1.49 (4H, *quint, J*6.8*, CH2*), and *δ*1.27 (6H, *m, CH2*) and 13-C (500 MHz, DMSO; *δ* ppm) as follows: *δ*174.9, *δ*34.1, *δ*28.9, and *δ*24.9. Figures 6–10 show proton nuclear magnetic resonance spectroscopy (^1^H NMR), heteronuclear single quantum coherence spectroscopy (HSQC), heteronuclear multiple bond coherence (HMBC), proton-proton correlated spectroscopy (COSY), and 13-carbon spectroscopy profiles of compound (2).

## 4. Discussion

Methanol was used at the initial stages of extraction as it is capable of extracting a large number of compounds at a wider range of polarity. This is the first time for both benzene-1,4-diol (1) (0.003%) and nonanedioic acid (2) (0.002%) to be isolated from the leaves and roots of *E. crassipes*, respectively. The isolation and purification of the two compounds were efficient as evident from the clean NMR spectra. Since DMSO was used in the spectroscopic experiments, its peak remains outstanding in both ^1^H NMR (*δ* 2.5) and 13-C (*δ* 40). The elucidation of both compounds was arrived at using a combination of spectroscopic techniques, and the proposed structures were confirmed with the masses of the compounds in the LCMS.

**Figure 11:**
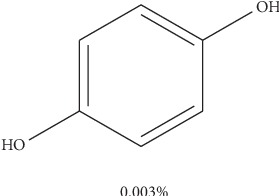
Proposed structure of compound (1): benzene-1,4-diol.

**Figure 12:**
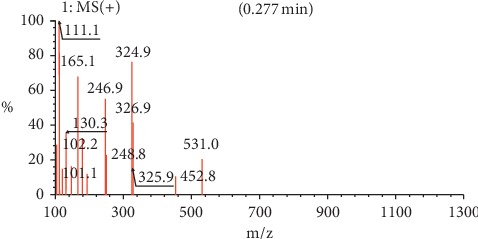
LCMS spectrum for compound (1).

For benzene-1,4-diol (1), 2 ^1^Hs appear at *δ*8.57 as shown in Figure 1, labelled as a and b for convenience of elucidation. The chemical environment they are in shows that they must be 2 hydroxy groups attached to an aromatic ring. A second chemical environment at *δ*6.56 has 4 ^1^Hs labelled c to f, meaning that they are all sharing the same environment in the aromatic ring as well. HSQC in Figure 2 shows that protons a and b are also attached directly to the same or two different carbons at *δ*150 but in the same region. The same carbon attachment is ruled out because an aromatic ring cannot have more than one proton attached to it, which confirms that there must be two protons attached to two different carbons but in similar chemical environments. Similarly, the four protons, c to f, also appear attached to carbons in the same chemical environment. HMBC indicates how the protons correlate with carbons 3 bonds away as shown in Figure 3, and Figure 4 shows that protons c to f are cosying with each other but not with a and b while a and b are also cosying with each other. The carbon 13 spectroscopy in Figure 5 confirms that the molecule has two distinct chemical environments as determined in the experiments described already. These experiments lead to a conclusion that the molecule is a symmetrical aromatic as shown in Figure 11 which was confirmed by the LCMS experiment in a positive mode to have a mass of 110 g/mol as shown in Figure 12.

**Figure 13:**
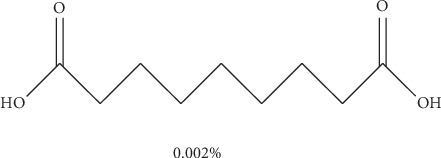
Proposed structure of compound 2: nonanedioic acid.

For nonanedioic acid (2), four regions appear in the ^1^H spectrum with 2 ^1^Hs (a and b) appearing in the N(CO)H or C(CO)H region. However, the next proton appears at *δ*2.1 as shown in Figure 6, which is more shielded upfield than it would have been if protons a and b were in an amide functional group. This confirms that the structure has a carboxylic acid group. These are followed by 4 ^1^Hs at *δ*2.1. These are 2 x CH_2_s and then followed by another set of 4^1^Hs at *δ*1.5 and lastly 6^1^Hs at *δ*1.2. This last group is tricky as they could be 3 x CH_2_s or 2 x CH_3_s. After trying several options, it was clear that CH_3_s could not make sense in the structure, which appears not to require terminal or branched methyl groups to be in line with the data given by its LCMS with a molar mass of 188 g/mol. These were therefore identified as CH_2_s, with two of them in the same chemical environment and one more in very close proximity to this environment, making their spectral presentation overlap (Figure 13).

**Figure 14:**
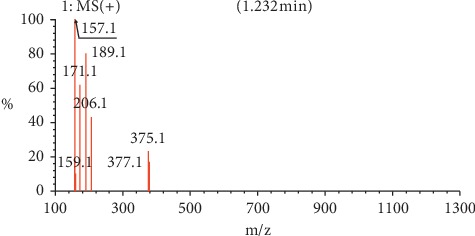
LCMS spectrum for compound (2).

HSCQ in Figure 7 showed that the carbon at *δ*50 does not have any protons attached to it, which means protons a and b should be attached to a hydroxyl group. HMBC confirms how the protons were linking with carbons 3 bonds away as shown in Figure 8, and COSY in Figure 9 indicates that protons a and b cannot be seen by any other proton which signifies that they are not neighbouring any proton in the molecule. COSY also shows that protons a and b cannot even see each other, and it shows that these two protons are not on the same carbon but are far from each other but in similar chemical environments.  Figure 10 solidifies that there are less number of chemical environments than the number that the integrated protons can be allocated to. This just shows that the structure should also be a symmetrical one with carboxylic acids at both ends, and the 6 protons of the 3 x CH_2_s described earlier can only make sense to be in the middle as suggested by the proposed structure in Figure 14. The LCMS profile of the compound confirms that the proposed structures are correct with a mass of 188 g/mol.

The molecule is polar and would be expected to have been washed off from the plant matrix considering that it is aquatic, but its presence in detectable amounts suggests that there was significant hydrogen bonding in the plant matrix or that it existed as fragments of its metabolites that had to undergo biosynthesis at a certain point and lastly, it would be existing as a larger complex structure that fragmented into smaller bits including (1). Though (2) would also exist in the roots of the plant as fragments or a complex that had to undergo some biosynthesis or fragmentation, there is a high chance that it would exist as it is surrounded by polar water molecules that would hardly wash it away from the plant matrix due to its nonpolar nature.

### 4.1. Drug Development Prospects of the Isolates

Benzene-1,4-diol (compound 1) and nonanedioic acid (compound 2) possess various bioactive properties. Benzene-1,4-diol (1) has anticancer activities against various cells [20]. Its derivatives such as hydroquinone 5-O-cinnamoyl ester and some alkyl and prenylated derivatives have also been reported to have cytotoxic activities against lung cancer [20–22], meaning that more optimization of the compound has a potential to develop more drug lead options against cancers. Nonanedioic acid (2) has antitumor properties in humans [23], antineoplastic and antileukemic activities [24], immunoregulatory and antiproliferative activities against cancer cells [25, 26], and cytotoxic activities and anti-inflammatory and antioxidant activities [27]. Some of its derivatives have also shown to be active against cancers [26] which give a good promise of its usefulness in drug development against cancers and other diseases.

Besides bioactivity, physicochemical characteristics of a chemical structure provide drug developers with enough pointers towards the ease or difficulty that will be associated with developing the molecules further into drugs [2, 28]. One good thing to note is that nonanedioic acid is not teratogenic and mutagenic and not toxic [23].  Table 1 shows that both molecules do not have AMES toxicity, ensuring that they would not be carcinogenic, and they are also not expected to be skin sensitizers. Both compounds do not have the ability to inhibit any cytochrome P450 enzymes, but compound (2) is a cytochrome P450 CYP2D6 substrate. The percentage absorption of the structures in human intestines is good enough (77% and 92% for 1 and 2, respectively), which would not require too high concentrations of drug material at the dose designing stage assuming they are being purposed for the same target.

Assessing the medicinal chemistry of the structures, the combination of a bulk aromaticity (nonpolar) with polar ends at opposite ends for compound (1) points to a good pharmacokinetic and pharmacodynamic balance if an oral drug is to be developed from this molecule. Its lipophilic aromatic region would enable its penetration through membranes, and the polar regions would facilitate its movement through aqueous phases and eventually making hydrogen bonding with target receptor proteins more effective [28]. On the other hand, compound (2), which is largely nonpolar, would not make a good oral drug as it would comfortably get stuck in membranes or have various nonspecific covalent binding which is known to enhance drug toxicity [7, 8, 28]. As such, there is need for further synthetic work to be done on compound (2) if it is considered for oral drug development. When bioactivity and the chemistry of the molecules are acceptable, other drug property assessments and adjustments need to be kept in check during the whole course of drug development.

## 5. Conclusion

Benzene-1,4-diol and nonanedioic acid have been isolated and purified from *E. crassipes* leaves and roots, respectively, for the first time, and their drug designing and development prospects are explained in detail. Knowing how important the plant is in its traditional medicinal use and the prospects of developing it further as potential hits into drugs and/or food supplements, the presence of these molecules gives a hint to what chemistry, pharmacological, and biological studies may need to be considered for optimization. *Eichhornia crassipes* should no longer be considered just as a menace weed but as one of the resources that, if well managed, can benefit the society through health management.

## Figures and Tables

**Table 1:** *In silico* drug pharmacokinetic and pharmacodynamic properties of the compounds.

Structural descriptor	Compound 1	Compound 2		Compound 1	Compound 2		Compound 1	Compound 2
	Value		Toxicity	Units/comment		Metabolism	Units/comments	
Molecular weight	110.112	188.223	AMES toxicity	No	No	CYP2D6 substrate	No	Yes
LogP	1.0978	1.8863	Max. tolerated dose in human (log mg/kg/day)	1.005	−0.171	CYP3A4 substrate	No	No
#Rotatable bonds	0	8	hERG I inhibitor	No	No	CYP1A2 inhibitor	No	No
#Acceptors	2	2	hERG II inhibitor	No	No	CYP2C19 inhibitor	No	No
#Donors	2	2	Oral rat acute toxicity (LD50)	1.909	1.722	CYP2C9 inhibitor	No	No
Surface area	47.02	77.57	Oral rat chronic toxicity (LOAEL) (mg/kg_bw/day)	2.781	3.147	CYP2D6 inhibitor	No	No
			Hepatotoxicity	No	No	CYP3A4 inhibitor	No	No
			Skin sensitisation	Yes	No			
			*T. pyriformis* toxicity (log ug/L)	−0.335	0.15			
			Minnow toxicity (log mM)	2.179	0.902			

Absorption	Compound 1	Compound 2		Compound 1	Compound 2		Compound 1	Compound 2
	Units/comment		Distribution	Units/comments		Excretion	Units/comments	

Water solubility (log mol/L)	−0.429		VDss in human (log L/kg)	−0.097	−1.241	Total clearance	0.55	1.478
Caco2 permeability (log Papp in 10^−6^ cm/s)	1.371	0.755	BBB permeability (log BB)	−0.258	−0.414	Renal OCT2 substrate	No	No
Human intestinal absorption (% absorbed)	76.754	91.905	CNS permeability (log PS)	−1.992	−3.008			
P-glycoprotein substrate	No	No						
P-glycoprotein II inhibitor	No	No						

## Data Availability

The data used to support the findings of the study are included within the article, and additional data if required could be obtained upon request.
